# A True View of Marriage

**Published:** 1892-08

**Authors:** 


### A TRUE VIEW OF MARRIAGE.

We copy the following article upon a subject of universal interest, from the *Progressive Age*.

Marriage in its completeness is a triune conjoinment: physical, mental-spiritual and celestial. The marriage of man and woman thus far has been merely physical; while spiritually they are living in a state of divorce. Their marriage is an animal marriage, and not a union of mind and soul. There can be no true marriage except as the man and woman are perfectly conjoined in these degrees; and there can be no real happiness or perfectly harmonious offspring. A house divided against itself cannot stand.

The disorder and discords of life are due to this divorcement of men and women in these higher phases of their nature. They are like the two sides of a triangle, that, instead of coming together above, to create the apex of power, are separated, and have no triune unity, that begins with the base and ends nowhere. And marriage cannot produce its true results until this unity of the triune elements in man and woman are conjoined.

It should be deeply impressed upon the mind, that the harmonious operation of all powers, from the greatest to the least, is due to the scientific observance of the laws that govern and create the power. All things, physical, mental-spiritual and celestial, operate according to well defined formulas, that can be investigated and analyzed. Nothing in the lower works arbitrarily; neither does it in the higher. There is no arbitrariness on any plane of evolution.—*World's Advanced Thought.*

